# Familial Mediterranean Fever in a 28-Year-Old Male Presented as a Painless Massive Pleural Effusion

**DOI:** 10.7759/cureus.41776

**Published:** 2023-07-12

**Authors:** George Dimeas, Ilias E Dimeas, Konstantina Papacharalampous, Eleftherios Chalvatzoulis, Zoe Daniil

**Affiliations:** 1 Department of Respiratory Medicine, University Hospital of Larissa, Larissa, GRC; 2 Pathology Department, IASO Thessalias General Hospital, Larissa, GRC; 3 Cardiothoracic Department, IASO Thessalias General Hospital, Larissa, GRC

**Keywords:** familial mediterranean fever, colchicine, mefv gene, periodic fever, video-assisted thoracoscopic surgery (vats), mediterranean fever, familal, fmf, pleural effusions

## Abstract

This case describes the first patient with familial Mediterranean fever (FMF) with massive left pleural effusion as the first clinical manifestation, to whom a video-assisted thoracoscopic surgery was performed to support the diagnosis. The patient was a 28-year-old male, who presented with dry cough and dyspnea but no fever. The lab findings showed hypoxemia (partial pressure of oxygen_ _= 65 mm Hg) accompanied by elevated inflammatory markers, including C-reactive protein at 7 mg/dl (<0.5 mg/dl), erythrocyte sedimentation rate of 46 mm/h (<20 mm/h), and serum amyloid at 56.7 mg/L (<10 mg/L). X-ray indicated the left pleural effusion was part of a bilateral recurrent painless pleuritis, as the right pleural thickening implied. Numerous biopsies were taken during the thoracoscopy, and the histopathology examination reported non-specific fibrous pleurisy. Colchicine administration, at first empirically for upcoming pericarditis, at the end was a significant clue for the diagnosis. Positive molecular testing for mutations in the familial Mediterranean fever (MEFV) gene contributed to the diagnosis of FMF, which was based on the Tel-Hashomer clinical criteria. The purpose of this article is hopefully to raise further awareness about patients with FMF presented with unusual manifestations of the disease.

## Introduction

Familial Mediterranean fever (FMF) is an autoimmune disease affecting people mostly in the Mediterranean area. Usual manifestations include recurrent fever along with serositis or arthritis. The disease is caused by a variation of mutations in the familial Mediterranean fever (MEFV) gene, which is located on chromosome 16 and specifically in exon 2 and exon 10 [1]. These mutations can be inherited in both an autosomal recessive and an autosomal dominant pattern. Usually, the first attack of the disease is presented before the age of 20 years. The diagnosis of FMF needs a clinical presentation of the disease and a genetic confirmation. According to the Tel-Hashomer clinical criteria at diagnosis, the patient must be presented with two major criteria or one major and two minor criteria.

Based on different clinical manifestations, three phenotypes of FMF have been described. Type 1 includes patients with acute onset of fever with classical symptoms, such as painful serositis and arthritis. In type 2, patients present only kidney amyloidosis with no fever or other symptoms. Type 3 is characterized by mutations of the MEFV gene without the classical clinical manifestations. According to these, our patient falls within type 3 [2].

Most of the time, the diagnosis of FMF can be really challenging in atypical presentations of the disease. An example of this can be cases of recurrent pleuritis as the sole manifestation, in which the time between the onset of disease and diagnosis was prolonged. In these cases, pleuritis is usually unilateral and resolves in a short time of hours or days, just like other forms of serositis leaving a pleural thickening as a subtle reminder of the inflammation. In cases with severe serositis, the patient is in danger of cardiac tamponade or respiratory distress due to the significant amount of effusion, either pericardial or pleural [3]. For the diagnosis of FMF, even a unilateral pleural effusion fulfills the criterion of febrile serositis. Therefore, the heterochronous bilateral pleural effusion of our case is a rarer manifestation that has been described only once in another case.

## Case presentation

**Figure 1:**
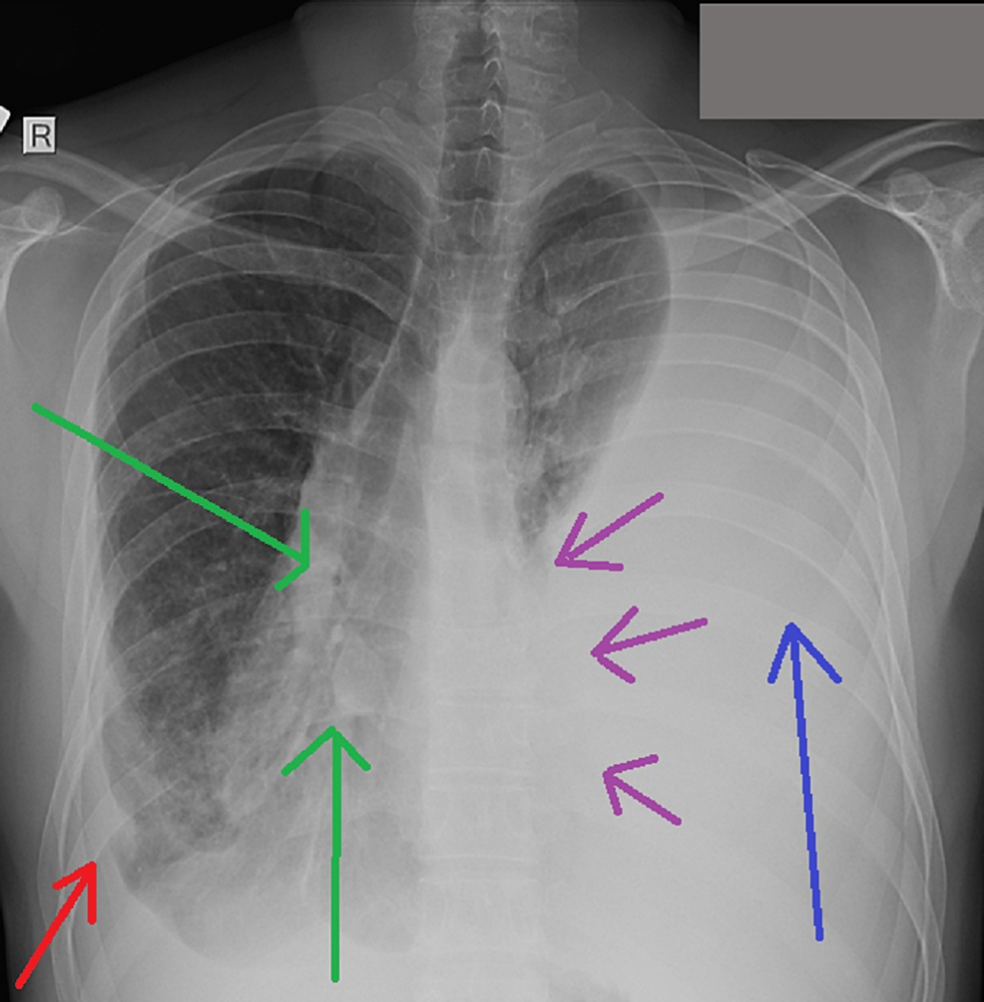
Admission chest X-ray showed a thickened pleura (red arrow) on the right side accompanied by a massive pleural effusion (blue arrow) on the left side. The cardiac silhouette is right-shifted (green arrows) with a blurred left side (purple arrows).

A 28-year-old non-smoker teacher without known medical history visited the emergency department of our hospital reporting dry cough and dyspnea that progressively worsened with onset 10 days ago. The patient did not report any weight loss or fever. He was neither on any medication nor had any allergies. No contact with asbestos was reported. Clinical presentation upon admission included tachypnea (respiratory rate of 23 breaths per minute) and respiratory distress. Lung auscultation revealed the absence of respiratory sounds on the left side. A chest X-ray (Figure 1) was performed, which showed a thickened pleura on the right side accompanied by a massive pleural effusion on the left side, which was confirmed with lung ultrasound and revealed an inversed diaphragm with paradoxical movement due to the amount of the effusion. The cardiac silhouette was right-shifted because of the aforementioned findings.

**Figure 2:**
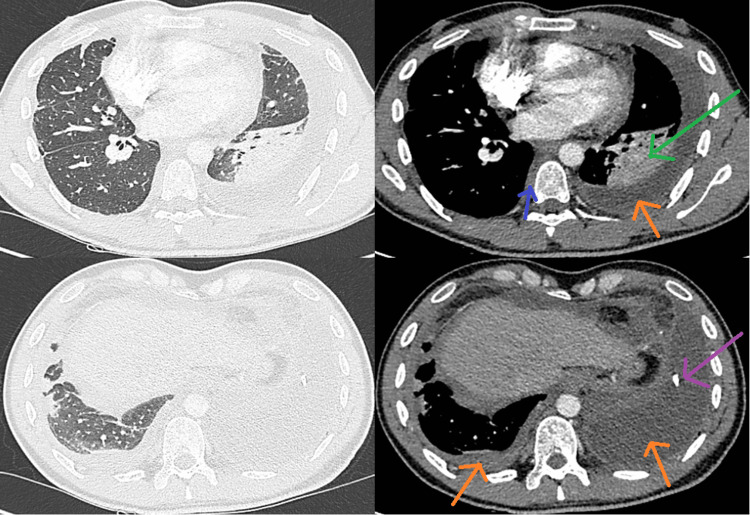
Chest computed tomography after evacuation of the pleural effusion showed bilateral small pleural effusions (orange arrows) with a right pleural thickening (blue arrow) and partial trapping of the left lower lobe (green arrow). A pleuro-catheter is noticed inside the left pleural effusion (purple arrow).

Heart sounds were normal and clear during the examination. No peripheral lymph nodes could be palpable. The patient was tachycardic (heart rate of 120 beats per minute) but hemodynamically stable. Arterial blood gas test showed hypoxemia with elevated alveolar-arterial gradient. Full blood cells count and biochemical panel were within normal range, except for the high values of acute phase proteins (Table 1). After a diagnostic thoracocentesis, pleural effusion was revealed to be a lymphocytic exudative with the values shown below (Table 2). Then a pleuro-catheter was inserted, through which 4.5 Lt of pleural effusion was evacuated. Periodic low afternoon fever waves up to 37.2°C were measured which were at first attributed to the thoracocentesis and the pleuro-catheter’s placement. Polymerase chain reaction (PCR) for acid-resistant bacteria and acid-fast stain were negative and the Mantoux test was measured at 0 mm. Moreover, a full serum autoimmunity panel for rheumatological diseases and a quantitative measurement of immunoglobulins (Ig) were normal. However, in the subclasses’ analysis, there was an elevation of IgG4. Chest computed tomography (CT) after evacuation of the pleural effusion (Figure 2) revealed bilaterally small pleural effusions, dominant on the left side with additional right pleural thickening without any other pathologic findings.

**Table 1:** Blood laboratory exams.

Test	Result	Normal values
Partial pressure of oxygen (Po_2_)	65 mm Hg	>60 mm Hg
Partial pressure of carbon dioxide (Pco_2_)	44 mm Hg	35-45 mm Hg
Arterial-alveolar gradient of partial pressure of oxygen (P_A-aO2_)	29.7 mm Hg	<9 mm Hg
C-reactive protein (CRP)	7 mg/dl	<0.5 mg/dl
Erythrocyte sedimentation rate (ESR)	46 mm/h	<20 mm/h
Serum amyloid	56.7 mg/L	<10 mg/L

**Table 2:** Pleural effusion laboratory exams.

Test	Result	Normal values
pH	7.34	>7.20
Lactate dehydrogenase (LDH)	190 U/L	135-225 U/L
Glucose	73 mg/dl	74-103 mg/dl
Total proteins	4.74 g/dL	6.4-8.3 g/dl
B-type natriuretic peptide (BNP)	124 ng/L	Not known
Adenosine deaminase (ADA)	7.9 U/L	<20 U/L
Effusion LDH to serum LDH ratio	0.95	-
Effusion proteins to serum proteins ratio	0.62	-
BNP of the effusion/BNP of the blood ratio	2.57	-

**Figure 3:**
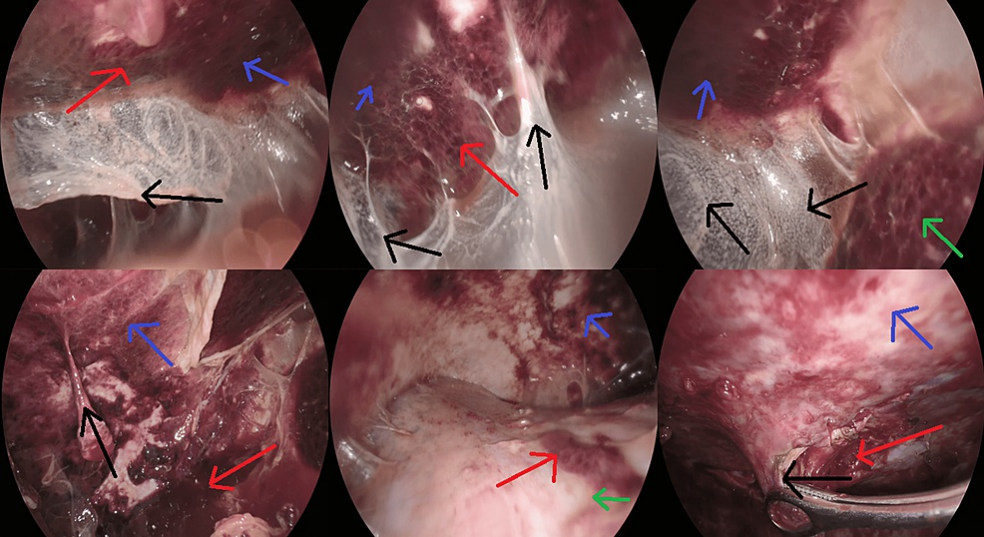
Thickened parietal (blue arrows) and visceral pleura (green arrows), spots with a film of old hemorrhagic material (red arrows), and thick white adhesions (black arrows) between the two layers, as shown during medical thoracoscopy.

**Figure 4:**
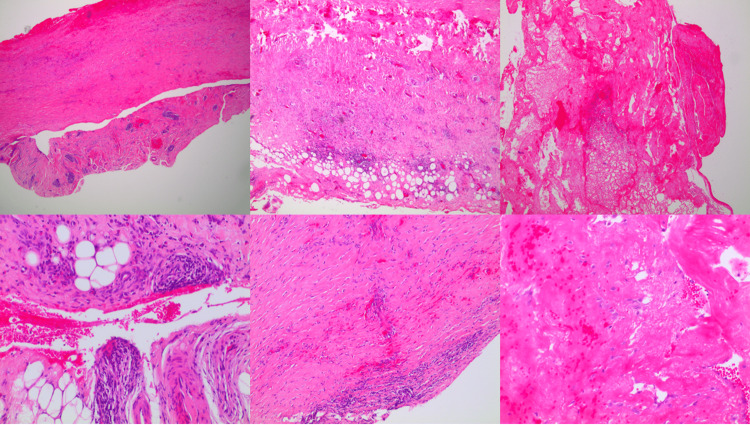
Pleura biopsies showing diffuse fibrosis, hyalinization, fibrin deposits, hemorrhage, and perivascular infiltrations consisting of lymphocytes and plasma cells, features consistent with the diagnosis of fibrinous nonspecific pleuritis.

A heart ultrasound was requested due to the bilateral effusions and was normal. An abdominal CT was also performed as a part of the hospital protocol for bilateral pleural effusion with normal cardiac function and it showed a small amount of ascitic fluid. Other tests such as CT of the neck, scrotum ultrasound, cancer markers, and immunophenotyping of the pleural effusion showed nothing irregular. Moreover, gastroscopy and colonoscopy were performed to exclude malignancy, which were both negative. Three cytologic examinations of the pleural effusion came back negative, so a medical thoracoscopy was programmed for the patient in the next few days. Pleuro-catheter was removed, and the patient got discharged with the instruction to keep track of his body temperature. As a result of the fever diagram, afternoon low-grade fever (maximum of 37.4°C) was confirmed and added to his symptoms, which was not previously noticed and reported by the patient. At the preoperative assessment in the readmission for the medical thoracoscopy, the pleural effusion showed to have been reproduced. Additionally, an emerging pericardial effusion was reported, therefore colchicine was initiated, and a re-evaluation was programmed after the invasive procedure. During the medical thoracoscopy, the pleural cavity was inspected with the presence of adhesions and loculations between the parietal and visceral pleura. It was important to start with the lysis of the adhesions, the breakdown of the loculations, and with lavage of the cavity. The inspection after showed that the parietal pleura and to some extent the visceral pleura of the basal segments were thickened and covered with a film of old hemorrhagic material (Figure 3). Numerous biopsies were taken from the posterior and lateral parietal pleura. The histopathology examination reported fibrosis, hyalinization, fibrin deposits, hemorrhage, and perivascular infiltrations consisting of lymphocytes and plasma cells, while the Congo red stain showed no amyloid deposits. The aforementioned morphologic features were consistent with the diagnosis of fibrinous nonspecific pleuritis (Figure 4). The aforementioned findings could be interpreted as non-specific fibrous pleurisy. Congo red and IgG4 stainings were negative for demonstration of amyloid or IgG4 in the tissue. No diagnostic signs of malignancy, mesothelioma, or other specific disease were detected.

**Figure 5:**
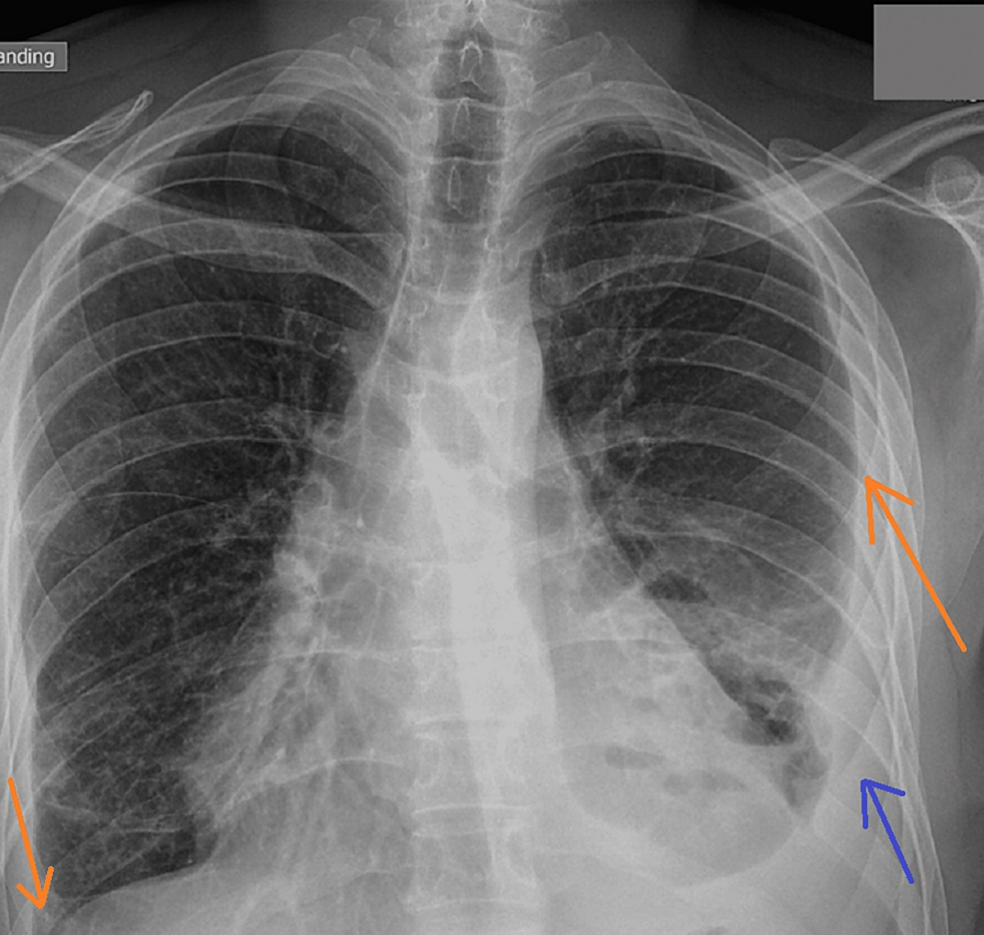
Almost no reproduction at follow-up after a month of the pleural effusion after the initiation of colchicine (blue arrow). Bilateral pleural thickening is noticed (orange arrows).

At re-evaluation after one month of colchicine, the dose was reduced by half. There was no pericardial effusion and the pleural effusion had not been reproduced (Figure 5). Because of this response, molecular testing for the entire coding system of the MEFV gene was requested. The diagnosis of FMF was reached based on clinical features combined with molecular confirmation.

## Discussion

This is probably the first case of atypical FMF in a patient with massive pleural effusion with an inverted diaphragm with paradoxical movement as the first manifestation of the disease where a video-assisted thoracoscopy was performed for the diagnosis and a B-type natriuretic peptide (BNP) fluid to serum ratio is reported, according to our knowledge.

The diagnosis of FMF in our case was reached based on clinical features and the Tel-Hashomer criteria (Table 3), combined with the confirmation of a heterozygous polymorphism R202Q/0 in exon 2 of the MEFV gene, which is already described in the Greek population [4].

**Table 3:** Tel-Hashomer diagnostic criteria for familial Mediterranean fever. The diagnosis is definite if two major or one major and two minor criteria are met. The diagnosis is probable if one major and one minor criterion are met.

Major criteria	Minor criteria
Recurrent febrile episodes associated with peritonitis, pleuritis, or synovitis	Recurrent febrile episodes
Amyloidosis of AA-type without a predisposing disease	Erysipelas-like erythema
Favorable response to daily colchicine	Positive history of familial Mediterranean fever in a first-degree relative

Regarding the differential diagnosis of our case, because of the lymphocytic predominance of the fluid and low adenosine deaminase (ADA), whole-body CT, gastroscopy, and colonoscopy were performed to exclude malignancy, and cytologic exams were negative. Chest and abdominal CTs did not show lymphadenopathy. For autoimmune diseases, a serum autoimmunity panel was sent, which was normal. Thyroid function was normal, and the diagnoses of Dressler syndrome or benign asbestos pleural effusion were excluded because of incompatible history or clinical presentation.

FMF is a clinical entity in which the diagnosis is based on clinical signs, especially the ones from the thorax. Video-assisted thoracoscopic surgery (VATS) is a minimally invasive procedure that can be a really useful tool to exclude other reasons from the differential diagnosis. It is especially helpful in cases with massive pleural effusions with a negative autoimmune panel and negative PCR for tuberculosis, like our case, in which the most probable diagnosis seems to be a malignancy despite the age of the patient. VATS is a minimally invasive procedure and can offer a lot of insight information to the doctor, as it offers a closer examination of lungs, pleura, and the chance to take biopsies with visual confirmation of possible lesions reducing the chances of false negative biopsies.

Recurrent pleuritis in our patient, shown by the right pleural thickening and left pleural effusion, in addition to the favorable response of the disease to colchicine, account for two major criteria for the diagnosis of FMF. Mutation testing that detected the polymorphisms in exon 2 in the MEFV gene confirmed the diagnosis.

Cases with sole pulmonary manifestations, such as painful pleural effusion and fever, have also been described [5-8]. However, in our patient, pleurisy and pleural effusion were not presented on both lungs simultaneously, as the right pleural thickening demonstrates. Additionally, the patient did not report any fever or pleurodynia in the past. These characteristics could be attributed to the FMF phenotype only with the pulmonary expression of the disease without systematic symptoms at the arising and later probable with polyserositis expressions, such as pericarditis and small ascites, as in our case.

FMF should always be considered in patients with atypical manifestations, even when fever or painful serositis is not present, to start treatment in the early stages. Molecular genetic confirmation should potentiate the diagnosis along with the established clinical criteria.

## Conclusions

Like in our case, FMF is not always presented in its typical form, so a high clinical index should alert the doctors in cases with atypical presentations. Especially in the Mediterranean region and in early ages, FMF should always be considered in the differential diagnosis of a patient with serositis. For the majority of cases with undiagnosed pleural effusions with negative cytologic examinations, medical thoracoscopy is necessary to establish the diagnosis. Additionally, as it is already known, both the response of the disease to colchicine administration and molecular testing remain important compounds for FMF confirmation.
